# Supplementary material for: MetaRibo-Seq measures translation in microbiomes
Source: Nat Commun. 2020 Jun 29;11:3268. doi: 10.1038/s41467-020-17081-z (PMC7324362; doi:10.1038/s41467-020-17081-z)
Supplement: Supplementary file 10 — Supplementary Data 7 [file 41467_2020_17081_MOESM10_ESM.zip › File2/Confidence_High_Taxonomy/1686_out.krona.html]

Javascript must be enabled to view this page.

members
magnitude
magnitudeUnassigned
count
unassigned
taxon
rank

1686\_out

3

2
superkingdom
3

3
phylum
1239

3
909932
class

3
909929
order

3
family
1843491

3
158846
genus

species
491921
3

SRS012902\_contig\_number\_905SRS023829\_contig\_number\_1583SRS075398\_contig\_number\_2685
